# Radiation-induced SLAMF1 reconfigures the SLAM family receptors landscape in triple-negative breast cancer cells

**DOI:** 10.7150/jca.137668

**Published:** 2026-09-18

**Authors:** Kyung-Hee Song, Jeong-In Park, Seung-Youn Jung, Hong-Jun Park, Jiyeon Ahn, Sang-Gu Hwang, Dae-Seog Lim, Jie-Young Song

**Affiliations:** 1 Division of Radiation Biomedical Research, Korea Institute of Radiological & Medical Sciences, Seoul 01812, Republic of Korea.; 2 Department of Biotechnology, CHA University, Gyeonggi-do 13488, Republic of Korea.

**Keywords:** SLAMF1, triple-negative breast cancer (TNBC), radiation therapy, immunotherapy, tumor microenvironment (TME)

## Abstract

Radiation therapy is a powerful immunomodulator capable of transforming the tumor microenvironment (TME). Signaling Lymphocytic Activation Molecule Family Member 1 (SLAMF1) is a cell surface receptor involved in various immune functions and cancer progression by regulating interactions with the immune microenvironment. However, the specific role of radiation in modulating the SLAM family within tumor cells remained poorly understood.

Ionizing radiation markedly increased the expression of SLAM family, including SLAMF1, and other immune checkpoints in three triple-negative breast cancer (TNBC) cell lines by flow cytometry analysis. Furthermore, dual immunofluorescence staining of breast cancer patient tissues confirmed that SLAMF1 is localized to tumor site independently of immune cells. Interestingly, SLAMF1 overexpression in TNBC cells distinctively reconfigures the expression of SLAMF2 and SLAMF5, both linked to immunosuppressive signaling.

These findings suggest a potential mechanism where radiation-induced SLAMF1 influences tumor immunogenicity by remodeling the inhibitory SLAM receptors landscape, potentially rendering tumor cells more susceptible to immune-mediated attack. As the first study to demonstrate endogenous SLAMF1 as a novel radiation-responsive factor in breast cancer, these results provide key insights for developing combined radiotherapy and immunotherapy strategies for TNBC.

## Introduction

Radiation therapy (RT) is one of the most common therapeutic modalities in oncologic treatment, traditionally valued for its ability to induce tumor cell death through direct DNA damage. However, emerging evidence highlights RT as a potent immunomodulator capable of transforming the tumor microenvironment (TME). In addition to local cytotoxicity, RT triggers the release of danger-associated molecular patterns (DAMPs) and tumor antigens, which in turn stimulate dendritic cells (DCs) and enhance antigen presentation to recruit activated CD8+ T cells 1, 2. Despite these immunostimulatory effects, RT can also elicit immunosuppressive responses such as the polarization of macrophages toward the M2 phenotype 3, neutrophils toward the N2 phenotype 4, and recruitment of myeloid-derived suppressor cells (MDSCs) 5, which may potentially limit the overall antitumor efficacy of RT.

The field of cancer immunotherapy has rapidly developed and established as an effective treatment for patients with advanced cancer, emerging as a potent therapeutic option alongside surgery, radiation, and chemotherapy. While immunotherapy has shown promising clinical outcomes, it is currently applicable to only a limited number of cancers 6. Among the different types of cancer immunotherapy, immune checkpoint inhibitors (ICIs) targeting PD-1/PD-L1 and CTLA-4 have fundamentally changed the landscape of treatment for advanced malignancies and offered the possibility of long-term and sustained therapeutic effects for some patients 7, 8. These agents function by disrupting inhibitory signals that induce T cell exhaustion and functional anergy, thereby restoring the cytotoxic potential of the immune system to recognize and eliminate cancer cells 9. Despite these remarkable clinical outcomes, a significant majority of patients exhibit primary or acquired resistance. In particular, many breast cancer subtypes are often classified as immunologically 'cold' tumors, characterized by a lack of tumor-infiltrating lymphocytes (TILs) and a highly immunosuppressive TME, which leads to suboptimal responses to existing therapies 10. While Triple-Negative Breast Cancer (TNBC) is considered the most immunogenic subtype of breast cancer, a significant proportion of patients still exhibit primary resistance to ICIs due to a complex, immunosuppressive TME. Thus, strategies to enhance the 'cold' or 'exhausted' immune state of TNBC through radiation are being actively explored. To overcome resistance in "cold" tumors, combined immunotherapy and RT treatments are being clinically attempted. Radiation can increase the susceptibility of tumor cells to immune-mediated death by promoting the release of tumor-specific antigens and regulating the expression of inhibitory or stimulatory checkpoints 11-13. For instance, the addition of RT to dual PD-1/CTLA-4 blockade has shown sensitization effect by stimulating the immune responses in patients with metastatic cancer 14, and the PACIFIC trial demonstrated significantly improved survival with adjuvant durvalumab (anti-PD-L1) after chemoradiotherapy 15. However, identifying the specific molecular mechanisms and interactions that regulate radiation- induced immune responses remains a critical necessity.

The signaling lymphocytic activation molecule (SLAM) family comprises nine type 1 transmembrane receptors (SLAMF1 to SLAMF9), which are predominantly expressed on diverse hematopoietic and immune cells 16-18. SLAM family receptors exhibit distinct ligand specificities and transmit signals to regulate a variety of immune responses, including the activation of T cells, B cells, NK cells, DCs and macrophages 16, 19, 20. While these receptors are well-known for their role in immune cells, recent studies have begun to reveal that SLAM molecules are also expressed endogenously within certain tumor cells, potentially acting as tumor-intrinsic factors that influence cancer progression 21. Most SLAM family receptors contain one or more immunoreceptor tyrosine-based switch motifs (ITSMs) in their cytoplasmic tails, which serve as docking sites for SH2 domain-containing adaptor proteins. The primary binding partners for ITSMs are SLAM-associated protein (SAP) and EAT-2, which transmit activating signals, or inhibitory phosphatases such as SHP-1, SHP-2, and SHIP-1, which attenuate downstream immune activation pathways 22, 23. Unlike most receptors, SLAM molecules often act as self-ligands through homotypic interactions, with the exception of SLAMF2 (CD48), which interacts heterophilically with SLAMF4 (CD244) 24. Despite their importance in immune modulation, the specific impact of radiation on the SLAM receptor landscape within tumor cells and how these changes alter the direct interaction between SLAMF receptors is not yet fully understood.

While ionizing radiation modulates multiple members of the SLAM family, functional prioritization requires evidence of clinical relevance in radiation-treated malignancies. In primary targets such as breast and lung cancers, high expression of SLAM family members (e.g., SLAMF2, SLAMF7, SLAMF8) does not consistently translate to clinical impact; only SLAMF1 maintains both significant tumor-specific expression and a consistent, statistically significant correlation with overall survival (OS). Consequently, SLAMF1 was selected as the primary subject of this investigation. In this study, we aim to investigate the tumor-intrinsic expression of SLAMF1 across three TNBC cell lines and sought to determine whether its expression is modulated by radiation. Furthermore, we examined how radiation-induced SLAMF1 reconfigures the expression landscape of other SLAM family members. Our findings provide a molecular basis for understanding the radiation-responsive SLAM receptor repertoire, suggesting a novel mechanism by which radiation alters the profile of immunomodulatory molecules in breast cancer cells.

## Materials and Methods

### Cell culture and irradiation

Human TNBC cell lines BT549, MDA-MB-231, and MDA-MB-468 were obtained from the American Type Culture Collection (Manassas, VA, USA). These cells were cultured in RPMI1640 medium (Welgene, Gyengsangbuk-do, Republic of Korea) supplemented with 10% fetal bovine serum (FBS, Welgene) and 100 U/mL penicillin/streptomycin (Welgene) at 37 °C under a 5% CO_2_ atmosphere. The human TNBC cells were exposed to radiation doses 12 Gy using ^137^Cs γ-source Biobeam 8000 irradiator (Gamma-Service Medical GmbH, Leipzig, Germany) at a dose rate of 3.5 Gy/min.

### Tissue microarray with Immunofluorescence (IF) staining

The breast cancer tissue microarray (TMA, #BR2082c) was purchased from TissueArray.Com LLC (Derwood, MD, USA) and comprised 192 individual tissue spots. Within this set, 192 tissue samples containing 10 distinct pathological subtypes were analyzed. Patient and tumor characteristics were sourced from the pathological reports provided by the manufacturer for subsequent analysis. The TMA slides were deparaffinized and rehydrated. The slides were boiled in citrate for antigen retrieval and blocked with 3% bovine serum albumin (GenDEPOT, Katy, TX, USA) for 1 h. Primary antibodies were incubated at 4 °C overnight: CD150 polyclonal antibody (#PA5-21123, Thermo Fisher Scientific, Waltham, MA, USA) and CD45 monoclonal antibody (#sc-28369, Santa cruz Biotechnology, CA, USA). On the next day, slide was incubated with goat anti-rabbit Alexa Fluor® 488 antibody (#A-11094, Invitrogen, Waltham, MA, USA) and goat anti-mouse Alexa Fluor® 594 Images, followed by DAPI nuclear counterstaining. Images were captured using an LSM880 confocal microscope (Carl Zeiss GmbH, Jena, Germany) and processed using the Zen 2.3 software (Zeiss).

### Flow cytometry

To analyze the expression of immune checkpoints and SLAM family receptors, TNBC cells were harvested and stained with the following anti-human monoclonal or polyclonal antibodies at a 1:200 dilution for 1 h at 4 ℃: FITC-CD274 (PD-L1, #393606), APC-CD273 (PD-L, #2345508), APC-CD365 (TIM1, #353906), APC-B7-H4 (VTCN1, #358108), FITC-VISTA (#378114), APC-CD155 (PVR, #337618), APC-CD270 (HVEM, #318808), FITC-CD150 (SLAMF1, #306303), FITC-CD70 (#355106), FITC-CD40 (#334306), PE-CD275 (ICOSL,#309404), PE-CD252 (OX40L, #326308), APC-CD137L (#311506), APC-CD48 (SLAMF2, #336714), PE-CD229 (SLAMF3, #326108), FITC-CD244 (SLAMF4, #393510), APC-CD84 (SLAMF5, #326010), PE-CD352 (SLAMF6, #317208), FITC-CD319 (SLAMF7, #331818) all purchased from BioLegend (San Diego, CA, USA). APC-GITRL (#FAB6941A) was obtained from R&D systems (Minneapolis, MN, USA). To exclude radiation-induced dead cell and non-specific artifacts, cells were harvested 24 h post-irradiation, washed with cold phosphate-buffered saline (PBS), and stained with Fixable Viability Dye (BioLegend) according to the manufacturer's instructions to discriminate dead cells. Data were acquired using the CyFlow® Cube6 flow cytometer (Sysmex-Partec GmbH, Görlizt, Germany) and subsequently analyzed with FlowJo software (v.10, Tree Star, Ashland, OR, USA).

### Establishment of a stable cell line

To establish SLAMF1-overexpressing breast cancer cells, the full length of SLAMF1 (RC223343, Slamf1 Human Tagged ORF Clone, NM_003037) or negative control scramble (PS100001, pCMV6-Entry, mammalian expression vector) was transfected into human breast cancer cell MDA-MB-231 and MDA-MB-468 using a lentivirus vector (Origene, Rockville, MD, USA). Briefly, 10 × 10^4^ cells were seeded into a 60 mm dish and medium containing Lipofectamine 3000 (Thermo Fisher Scientific) and a lentiviral vector was added the following day. The next day, a total of 1 μg/ml neomycin (Invivogen, San Diego, CA, USA) was used following culture for 3 days to select the stable cell lines. Following the selection, the cells were cultured in a standard neomycin-containing medium, confirming mRNA and protein expression levels.

### Statistical analysis

GraphPad Prism software version 8 (GraphPad, La Jolla, CA, USA) was used to perform statistical analyses. All data are expressed as mean ± standard error of the mean. Analysis of variance and Tukey's post-hoc test were performed to determine significant between-group differences.

## Results

### Ionizing radiation altered immune checkpoint expression in breast cancer

We first evaluated the radiation-induced alterations in immune checkpoint expression across three BT549, MDA-MB-231, and MDA-MB-468 breast cancer cell line following 12 Gy irradiation, and performed flow cytometry analysis. A single dose of 12 Gy was chosen to model hypofractionated stereotactic body radiation therapy, as 8-12 Gy falls within the optimal window to activate the cGAS-STING pathway without inducing TREX1 25, 26. Furthermore, our previous study confirmed that 12 Gy significantly enhances SLAMF1 expression compared to 3 Gy in TNBC cells without inducing marked cell death within 48 h 27. High-dose irradiation significantly and consistently upregulated the induction of immunosuppressive ligands including PD-L1, TIM-1, and PVR across all three TNBC cell lines. Other inhibitory markers displayed cell line-dependent response. PD-L2 and VTCN1 were significantly upregulated in MDA-MB-231 and MDA-MB-468 cells, whereas VISTA exhibited an increasing pattern without reaching statistical significance in any of the three cell lines. Of note, among the immunostimulatory checkpoints, SLAMF1, CD70, and CD40 were also found to be upregulated (Fig. 1). These findings provide new evidence that irradiation can be a key driver of immune checkpoint regulation and activation at the plasma membrane of breast cancer cells, implying that these cell-surface alterations may potentially modulate intercellular immune interaction within the TME.

### SLAMF1 was a tumor-intrinsic factor in breast cancer

Although SLAMF1 is generally known to be expressed in hematopoietic and immune cells, our previous results suggested that SLAMF1 was also present in breast cancer and other types of tumors 27. To verify this, we performed dual-immunofluorescence staining using SLAMF1 and the pan-leukocyte marker CD45 on tissue microarrays (TMAs) slide. SLAMF1 staining was observed to have lower fluorescence intensity in normal breast tissue compared to malignant lesions. Pathologically, high expression of SLAMF1 was confirmed across a broad spectrum of breast cancer subtypes, including metastatic, invasive, and squamous cell carcinoma in situ. To confirm that SLAMF1 expression does not originate from immune cells within the tumor, co-staining with the pan-leukocyte marker CD45 was performed. As a result, it was observed that SLAMF1 was expressed not only in immune cell-rich regions but also in CD45-negative compartments of the tumor (Fig. 2). These results demonstrated that SLAMF1 is expressed in breast cancer cells themselves, independently of hematopoietic and immune cells.

### Ionizing radiation induced differential expression of SLAM family receptors in breast cancer

Next, the effect of ionizing radiation on the expression profile of SLAM family members (SLAMF1-7) in TNBC cells was investigated using flow cytometry. The basal expression levels of most SLAM family members were relatively low in all TNBC cells, but SLAMF5 and SLAMF2 showed relatively high expression levels. Irradiation induced significant upregulation of SLAM family receptors in all three TNBC cell lines (BT549, MDA-MB-231, and MDA-MB-468) compared to the non-irradiated controls (Fig. 3). In particular, the result that radiation increased the expression of not only SLAM family members but also various immune checkpoints as shown in Fig. 1 suggests that radiation can actively reconstruct the molecular environment on the surface of tumor cells. These findings demonstrate that radiation-responsive SLAM receptors can play a specific role in regulating immune cell activity by maintaining a balance between activation and inhibition signals on tumor.

### High expression of SLAMF1 affects the expression of other SLAM family receptors

Considering the significant positive prognostic value of SLAMF1, -2, and -3 in breast cancer patients and SLAMF1 in lung cancer patients, we aimed to identify the specific role of SLAMF1 as a radiation response factor. To evaluate the crosstalk among SLAM family receptors, we established stable SLAMF1-overexpressing breast cancer cell lines. In MDA-MB-231 cells overexpressed with SLAMF1, the expression of SLAMF2 and SLAMF5 was reduced, while the expression levels of other SLAMF subtypes remained unchanged (Fig. 4A). In MDA-MB-468 cells, overexpression of SLAMF1 significantly reduced the expression of most SLAMF members, excluding SLAMF4 and SLAMF7 (Fig. 4B). These results suggested that SLAMF1 was identified as a key radiation-responsive factor that, when upregulated, reconfigures the expression landscape of other SLAM members.

## Discussion

Recently, considerable efforts have been devoted to elucidating the functions of the immune system within the complexity of the TME and to harnessing immune cells for effective tumor eradication. The discovery of immune checkpoint mechanisms has revolutionized our understanding of immune regulation and enabled the development of groundbreaking therapeutic interventions 28. Among these, PD-1 ligands (PD-L1 and PD-L2), expressed by both malignant cells and APCs in the TME, interact with PD-1 on activated T cells to induce exhaustion, anergy, and apoptosis 29, 30. This suppresses the activity of cytotoxic T cells, promoting immune evasion and tumor progression. PD-L1 inhibitors such as atezolizumab and durvalumab have demonstrated clinical benefits in NSCLC, urothelial carcinoma, and TNBC. However, therapeutic efficacy is not universal across patients 31-33.

Under these circumstances, we aimed to identify immunomodulatory molecules that are altered by radiation in tumors to overcome these therapeutic limitations. SLAMF1 belongs to the SLAM family of hematopoietic co-receptors, whose functional output is determined by the intracellular adaptor SAP. In SAP-expressing cells, SLAMF1 typically transmits co-stimulatory signals that promote T cell activation and differentiation, whereas in the absence of SAP, it can undergo a functional switch to deliver inhibitory signals. Unlike canonical inhibitory immune checkpoints such as PD-L1 or PVR, SLAMF1 exerts broader immunomodulatory functions mediated through this SAP-dependent signaling axis.

As shown in Figure 1, ionizing radiation significantly increased the expression of both immunosuppressive and immunostimulatory checkpoints in three TNBC cell lines, suggesting that RT actively modulates a broad spectrum of immune-related factors in tumors. Although RT can activate immune evasion molecules such as PD-L1 and PVR, blockade of these checkpoints synergizes with RT to enhance cytotoxic T cell activation and tumor cell death 34, 35. Conversely, radiation-induced increases in immunostimulatory factors may enhance tumor immunogenicity. For instance, CD137L induction in melanoma cells has been shown to promote CD8+ T cell survival and augment antitumor immunity both *in vivo* and *in vitro* 36. Previous studies have shown that high expression of SLAMF1 enhances the antitumor effect by activating T cell function in xenograft models. SLAMF1 is expressed in several cancer types and shows a positive correlation with improved survival in breast cancer 27. Therefore, radiation-induced immunostimulatory molecules may reinforce the intrinsic immunogenicity of the tumor and its microenvironment.

We further confirmed SLAMF1 expression in breast cancer patient tissues. Given its abundant presence in immune cells, dual immunofluorescence staining was employed to distinguish tumor specific expression. SLAMF1 partially overlaps with CD45-positive lymphocytes, but it has also been observed to be expressed in CD45-negative tumor sites (Figure 2). These findings provide clinical evidence that SLAMF1 is endogenously expressed in solid tumors, beyond its conventional role in immune cells. Nevertheless, the mechanisms underlying SLAMF1 and other SLAM family members in breast tumors remain incompletely understood.

In addition to SLAMF1, ionizing radiation increased the expression of other SLAM family receptors in breast cancer cell lines (Figure 3). The basal expression levels of SLAMF2, SLAMF5, and SLAMF6 were relatively high, and radiation increased the expression of most SLAMFs. However, the degree of induction after radiation was similar among the SLAM family members, meaning that this alone does not necessarily reflect therapeutic or prognostic importance. Crucially, our analysis of clinical databases for breast and lung cancer demonstrated that SLAMF1 is a unique factor in predicting OS. Our recent functional studies demonstrated that SLAMF1 overexpression in tumor cells enhances CD8^+^ T cell infiltration and reduces tumor growth *in vivo*, consistent with immune-mediated anti-tumor effects 27. Previous studies have implicated that these receptors are associated with breast cancer. SLAMF2 (CD48) expression is upregulated and positively associated with favorable clinical outcomes in patients with breast cancer 37. In contrast, the expression of SLAMF4 (CD244) in TNBC is lower than that in healthy breast tissue, which correlates with a poorer prognosis and decreased survival 38, 39. SLAMF5 expression is elevated in TNBC patients, and its level correlated with increased immune suppression and poor prognosis 40. SLAMF6 has been identified as a core prognostic gene associated with improved survival in TCGA-based breast cancer analyses 41, 42. Our study is the first to report that radiation significantly induces SLAMF expression in breast cancer, suggesting molecular reorganization of the tumor surface and highlighting SLAMFs as predictive therapeutic targets.

Although SLAMF1 is endogenously expressed in several tumors, its role in tumor progression and anti-tumor immunity has not been fully elucidated. Bologna *et al*. demonstrated that an agonistic SLAMF1 antibody induces autophagy and suppresses disease progression in aggressive chronic lymphocytic leukemia (CLL) cells by inhibiting CXCL12-dependent chemotaxis 43. Similarly, Von Wenserski *et al*. reported that SLAMF1 or SLAMF7 overexpression in CLL cells impairs B cell receptor signaling and sensitizes leukemia cells to NK cell-mediated surveillance 44. In the present study, SLAMF1 overexpression consistently downregulates SLAMF2 and SLAMF5 in breast cancer cells (Figure 4). SLAMF2 contributes to immunosuppression in regulatory T cell and NK cells in hepatocellular carcinoma and renal cell carcinoma 45, 46. Besides, SLAMF5 promotes immunosuppression by driving MDSC accumulation and enhancing PD-L1 and PD-1 signaling in hematological malignancies 47, 48. Thus, SLAMF1-mediated suppression of SLAMF2 and SLAMF5 provides a novel mechanistic rationale for further investigating the combination of RT and immunotherapy to modulate tumor-induced immunosuppression.

It is well appreciated in tumor immunology that the balance between activating and inhibitory co-signals on tumor cells is a critical determinant of immune cell activation and anti-tumor immunity 49, 50. In this context, radiation-induced SLAMF1 upregulation alters the landscape of cell-surface co-signaling molecules, including other SLAM family receptors, raising the possibility that these tumor-intrinsic receptor alterations may influence functional signals to interacting immune cells within the microenvironment. However, as our current data are limited to receptor expression profiling without direct functional immune assays, these findings should be interpreted as a mechanistic hypothesis that receptor landscape changes serve as a potential driver of immune regulation, warranting future functional validation.

In conclusion, we demonstrated that SLAMF1 is expressed in breast tumor tissue and infiltrating immune cells, and that ionizing radiation significantly upregulates SLAMF1 along with other SLAM family receptors and immune checkpoints. Elevated SLAMF1 suppresses inhibitory SLAM receptors, thereby potentially rendering breast cancer cells more susceptible to immune-mediated attack. Radiation-induced SLAMF1 may thus serve as a crucial factor in enhancing tumor immunogenicity. However, several questions remain: how SLAMF1 interacts with other immune checkpoints, why distinct responses occur among SLAM family members, and which factors determine their functional relevance. Future studies should investigate which immune cells radiation-induced SLAMF1 recruits and how it activates them to promote tumor death. Ultimately, correlating SLAMF1 expression with the efficacy of ICIs in patients receiving RT will be essential for developing personalized radiation-immunotherapy strategies.

## Figures and Tables

**Figure 1:**
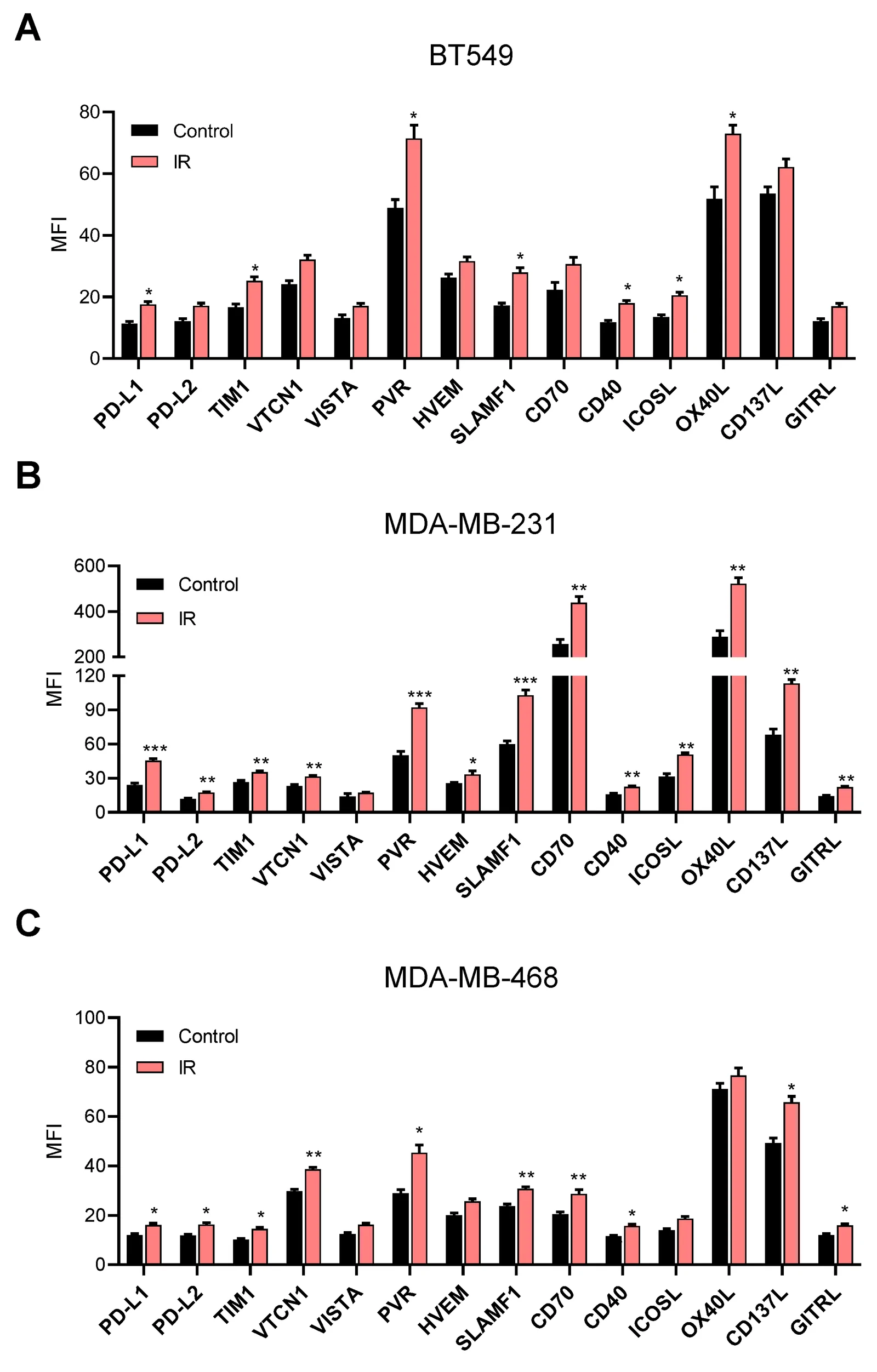
Ionizing radiation-induced expression of SLAMF1 and immune checkpoints in TNBC cell lines. Three TNBC cell lines-(A) BT549, (B) MDA-MB-231, and (C) MDA-MB-468-were subjected to 12 Gy of irradiation. The cell surface expression of SLAMF1, along with inhibitory and stimulatory immune checkpoints, was analyzed by flow cytometry after 24 h. Data represent the mean ± SEM of three independent experiments. Statistical significance is indicated as * *p* < 0.05, ** *p* < 0.01, *** *p* < 0.001. vs Control.

**Figure 2:**
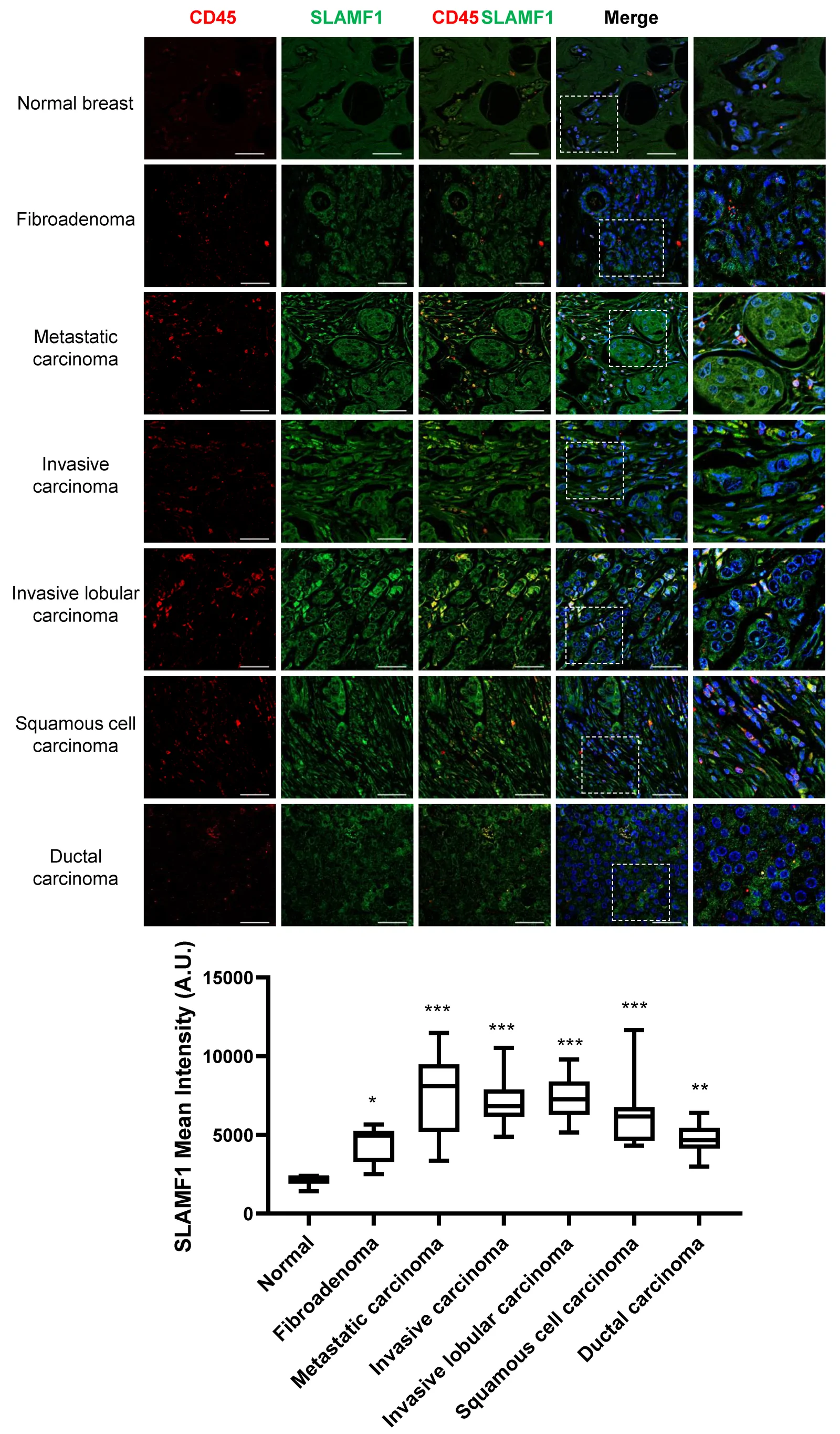
Differential expression of SLAMF1 and CD45 across human breast tissues. Representative immunofluorescence images of tissue microarrays (TMAs) containing normal breast tissue, a benign fibroadenoma lesion, and five malignant subtypes of breast cancer. Sections were stained for SLAMF1 (green) and the pan-leukocyte marker CD45 (red), with nuclei counterstained using DAPI (blue). Quantitative analysis of SLAMF1 expression across histological subtypes was determined by mean fluorescence intensity (MFI) per tissue core. Scale bars = 50 μm. Statistical significance is indicated as * *p* < 0.05, ** *p* < 0.01, *** *p* < 0.001. vs Normal.

**Figure 3:**
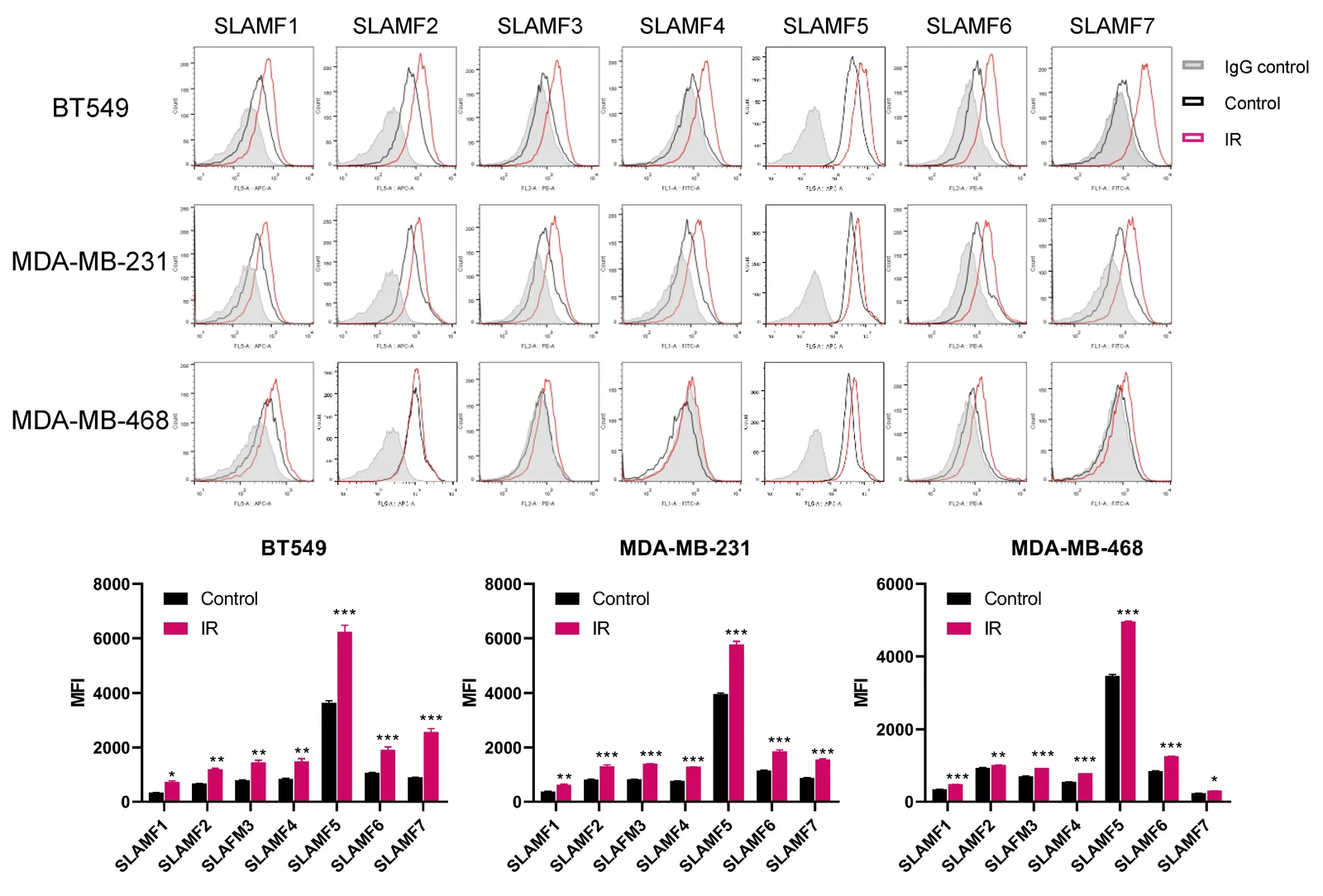
Alteration in SLAM family expression following irradiation in TNBC cell lines. Three TNBC cell lines BT549, MDA-MB-231, and MDA-MB-468 were irradiated with 12 Gy, and the level of SLAM family members (SLAMF1-7) were analyzed by flow cytometry after 24 h. Data represent the mean ± SEM of three independent experiments. Statistical significance is indicated as * *p* < 0.05, ** *p* < 0.01, *** *p* < 0.001. vs Control.

**Figure 4:**
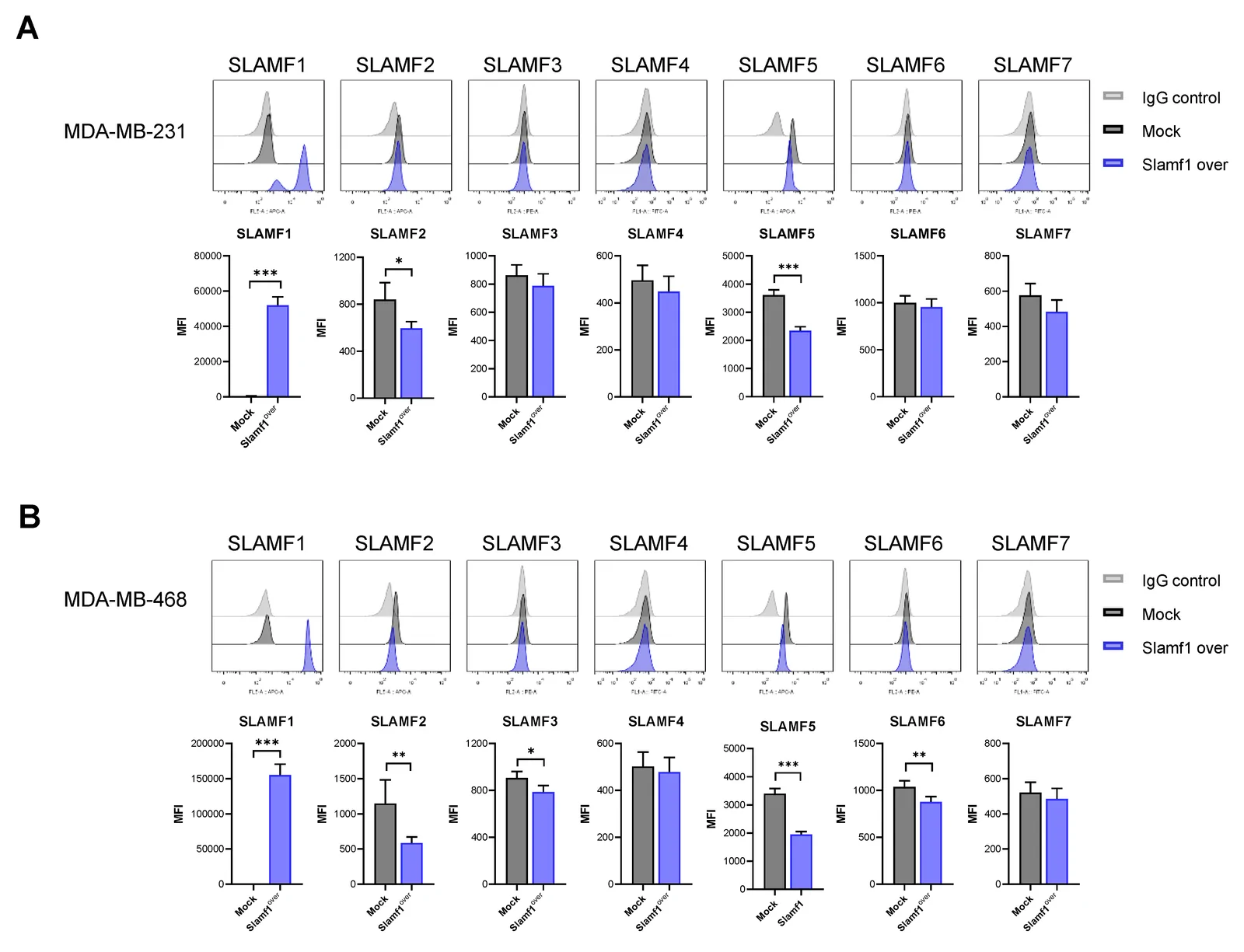
SLAM family expression profiles in SLAMF1-overexpressing breast cancer cells. Stable SLAMF1 overexpressing (A) MDA-MB-231 and (B) MDA-MB-468 cells were analyzed for the expression of SLAM family members using flow cytometry. Data represent the mean ± SEM of three independent experiments. Statistical significance is indicated as * *p* < 0.05, ** *p* < 0.01, *** *p* < 0.001. vs Mock.

## Data Availability

The datasets used and/or analyzed during the current study are available from the corresponding author on reasonable request.
